# A Comparative Evaluation of the Sedative Effects of Nitrous Oxide-oxygen Inhalation and Oral Midazolam-Ketamine Combination in Children

**DOI:** 10.5005/jp-journals-10005-1547

**Published:** 2018-10-01

**Authors:** Ilasrinivasan, Jyothsna V Setty, Shyamachalam, Priya Mendiretta

**Affiliations:** 1 Professor and Head, Department of Pedodontics and Preventive Dentistry, MR Ambedkar Dental College and Hospital, Bengaluru, Karnataka, India; 2 Professor, Department of Pedodontics and Preventive Dentistry, MR Ambedkar Dental College and Hospital, Bengaluru, Karnataka, India; 3 Ex-resident, Department of Pedodontics and Preventive Dentistry, MR Ambedkar Dental College and Hospital, Bengaluru, Karnataka, India; 4 Ex-resident, Department of Pedodontics and Preventive Dentistry, MR Ambedkar Dental College and Hospital, Bengaluru, Karnataka, India

**Keywords:** Anxiolysis, Nitrous oxide-oxygen inhalation, Oral ketamine-midazolam combination, Sedative effects.

## Abstract

**Aims:**

To compare nitrous oxide-oxygen inhalation and low dose oral midazolam-ketamine combination for anxiolysis in the management of children aged between 3 to 10 years for dental treatment.

**Materials and methods:**

A comparative clinical study with equal number of subjects in both the groups evaluating efficacy of oral ketamine-midazolam combination and nitrous oxide-oxygen inhalation in children with Frankl behavior rating score 2 and ASA1.

A total of 30 children were equally divided into 2 groups, oral midazolam-ketamine (MK) group which received 0.25mg/ kg midazolam with 3mg/kg ketamine in combination and the Nitrous oxide-oxygen (N) group which received nitrous oxide-oxygen inhalation. The parameters evaluated were the drug/ mask acceptance, need for the use of a physical restraint. Houpt’s sedation scale, faces pain score, sedation duration, time taken to achieve the maximum sedation and adverse reactions were assessed. Student t-test was used for comparison between the groups and proportions were compared using Chi-square test.

**Results:**

The results found no statistically significant differences between the groups in all the parameters except for the duration of sedation and the time taken to achieve maximum sedation which were higher in oral MK group than the Nitrous-oxide oxygen inhalation group.

**Conclusion:**

Both oral-midazolam and ketamine combination and nitrous oxide-oxygen inhalation sedation were found to have similar clinical success among 3 to 10-year-old children in bringing about anxiolysis during dental treatment.

**Clinical Significance:**

Both oral ketamine-midazolam combination, nitrous oxide-oxygen inhalation are equally effective for anxiolysis in children during dental treatment.

**How to cite this article:** Ilasrinivasan, Setty JV, Shyamachalam, Mendiretta P. A Comparative Evaluation of the Sedative Effects of Nitrous Oxide-oxygen Inhalation and Oral Midazolam-Ketamine Combination in Children. Int J Clin Pediatr Dent., 2018;11(5):399-405.

## INTRODUCTION

Management of uncooperative children for dental treatment is a challenging task. Providing pain control and anxiety reduction with psychological techniques may not prove fruitful at all times, necessitating the need to use pharmacological approaches for the same. In children, analgesia/anxiolysis may expedite the treatment procedure and ensure limited unpleasantness. Inadequate management of patient’s procedural pain and distress not only adversely affects experience and attitude towards dentistry, but also impacts treatment outcomes. Sedation is a pharmacological technique with a relatively safe and effective way to facilitate dental care in anxious patients.^1^

Conscious sedation, an anxiety control technique for a pediatric patient in dental office has been advocated by the American Academy of Pediatric Dentistry (AAPD) in 2012, usually employed for management of extremely anxious children.^2^ Presently termed as moderate sedation or sedation analgesia, AAPD now defines it as a drug-induced depression of consciousness during which patients respond purposefully to verbal commands.^3^ Conscious sedation in medicine is used as a premedication prior to the administration of general anesthesia wherein the goal is to have the child arrive in the operating room calm and quite with intact cardiopulmonary reflexes.^4,5^ However, in the dental scenario, administration of a local anesthetic agent, use of a low-speed drill, constant vibration, continuous suction, superimpose stimulations which predispose a child to retain a greater alert response state.^6^ The aim, therefore, is to provide optimal sedation and anxiolysis and at the same time to successfully control the child’s behavior.

An ideal pediatric dental sedative should be safe, cause minimum respiratory depression, provide adequate sedation, should bring about minimal patient movement, have an early onset of drug action and provide an adequate working time.^7^ The literature documents the use of various sedative agents and combinations (such as-short-acting benzodiazepines, barbiturates, inhala-tional agents, opioids) but none could be termed as an ideal sedative agent for children.^7^ Sedative drugs may be administered by oral, inhalation, rectal, submucosal, intramuscular, or intravenous routes. The selection of the technique depends on the clinician’s choice.

Oral sedation is the oldest known, yet effective, economical and the most accepted of all routes of conscious sedation.^8^ Midazolam, when administered alone orally, is also a popular sedative agent but its reported efficacy is only 60 to 76%.^9^ Ketamine is a drug with a bioavailability of only 16% when administered orally. In low dosages, it produces variable anxiolytic results and in higher dosages is associated with psychomimetic and sympathomimetic side effects.^10^ The use of a combination of ketamine and midazolam in conscious sedation for oral administration was first described by Lin, Moynihan, and Hackle.^11^ The combination of midazolam and ketamine when administered orally maintains the anxiolysis provided by mid-azolam and adds the sedative and analgesic properties of ketamine, while reducing the undesirable side-effects. This drug combination maximizes the sedation level obtained while keeping low drug dosages with results better than those when administered alone.^12^

In the recent past Pediatric dentists have recognized the utility and comfort of nitrous oxide-oxygen inhalation sedation to reduce pain and improve behavior during dental treatment.^13^ The use of inhalation sedation has been well documented in the literature, where administration of nitrous oxide is 50% or less with the balance as oxygen, without any other sedative, narcotic, or another depressant drug before or concurrent with the nitrous oxide to an otherwise healthy patient in ASA I and ASA II.^3^

As there is limited literature comparing oral mid-azolam and ketamine combination with nitrous oxide-oxygen inhalation sedation in children, the present study was carried out in the age range of 3 to 10 years in whom early dental problems can be tackled with the use of the same.

## MATERIALS AND METHODS

After obtaining ethical clearance from the Institutional Review Board and Ethics Committee, an informed oral and written consent was obtained from the parents of children selected for study, belonging to Frankl’s behavior rating score 2 and ASA1. Systematic random sampling was done based on the registration of the patients to the Pediatric Dental Department fulfilling inclusion and exclusion criterion until the desired sample size of 15 children in each group was met. Student t-test was used for comparison between the groups and proportions were compared using the Chi-square test.

### Inclusion Criteria

- Children aged between 3 to 10 years
- The patient should belong to ASA1.
- The patient is depicting a negative (score 2) as on Frankl’s behavior rating scale.
- The patients for whom basic behavior guidance techniques have not been successful.
- The patients are undergoing dental procedures like extraction and endodontic treatments requiring the administration of a local anesthetic and duration of procedure duration not exceeding one hour.

### Exclusion Criteria

- Children who were not cleared by the anesthetist for the sedation procedure.
- Any known allergy or hypersensitive reaction to the drugs being used throughout the procedure.
- Children who were administered analgesics six hours prior to the procedure.
- Children who were recently administered medications such as erythromycin or anticonvulsants which may interfere with the pharmacokinetics of midazolam.

*Group* N-children received nitrous oxide-oxygen inhalation at a concentration below 50%

*Group* MiOchildren, received oral midazolam (0.25 mg/ kg) and oral ketamine (3mg/kg) combined with a mango drink (FROOTI^®^, Parle Agro Pvt Ltd., India)

The patients were requested to follow the pre-procedural guidelines,^14^ and all of them were examined on the day of the sedation procedure by the anesthetist for medical clearance. The weight of the child along with the blood pressure, heart rate and oxygen saturation at baseline was also recorded. The anesthetist monitored the patients throughout the procedure until discharge.

### Group N

A “tell show do” approach was used where the child was explained about the placement of nasal mask over the nose for inhalation of a cold air followed by a sweet smelling breath.

Once the child was ready, an appropriately sized nasal hood was selected, and the oxygen cylinder was turned on. The acceptance of the mask by the child was assessed at this stage. The flow rate was adjusted after observing the reservoir bag. Initially, 100% oxygen was administered for 5 minutes following which nitrous oxide was slowly introduced, 10% at every 5-minute intervals as per the patients need without exceeding the concentration over 50%. The signs for sedation onset were observed, i.e. drooping of the eyelids, tingling sensation in the extremities. Once the signs were evident, the procedure was started. Nitrous oxide was maintained at this level with a gradual decrease in the concentration as the procedure approached termination, when 100% oxygen was administered for 5 minutes, and then the nasal mask was removed.^3^

### Group MK

Vials of 5 mL midazolam (MEZOLAM 1 mg/mL, Neon Laboratories Ltd, Bengaluru, India) and 10 ml ketamine (ANEKET 50 mg/mL, Neon laboratories, Bengaluru, India) were used in the study. The drug dose was measured according to the weight of the child. The drug was drawn from the vial using a 27 gauge disposable needle for accuracy. Once both the drugs were drawn and transferred into a disposable cup which contained the flavored fruit drink (Frooti^®^, Parle Agro Pvt Ltd., India), drug acceptance of the child was assessed. If the child expectorated all or part of the drug, a reappointment was scheduled for the same.

The time of drug administration was noted, and the child was under observation by the anesthetist. A minimum of 30 minutes waiting period was given between the drug administration and parental separation to facilitate the onset of drug action.

The signs of sedation onset, i.e., dazed look, delayed eye movement, lack of muscle coordination; slurred speech and sleep were assessed. After parental separation was easily achieved, the procedure (either extraction/ pulpectomy) was carried out under local anesthesia. The effectiveness of the sedative agent was assessed using Houpt’s sedation scale (involves sleep, movement, crying and overall behavior)^15^ and patient was discharged only when a score of 10 was obtained as per the Alder-ate Recovery Score(involves activity scores, respiration, circulation, consciousness, color).^8^ The clinician from the operatory was in contact with the parents of the child for 24 hours to determine any undesirable side effect like vomiting, sleep pattern, and alertness of the child.

## RESULTS

The present study evaluated 0.25mg/kg of oral mid-azolam in combination with 3mg/kg of ketamine compared to nitrous oxide-oxygen inhalation for conscious sedation in 30 children aged between 3 to 10 years. The sedation efficiency, duration, the need for the use of physical restraint and the time taken to reach maximum sedation were assessed.

The studied sample consisted of five (33.3%) males and 10 (66.7%) females in the MK group with a mean age of 5.4 years (± 1.81). Nitrous oxide group consisted of 6 (40%) females and 9 (60%) males with an average age of 5.9 (± 1.67) years with both the variables not being statistically significant in both the groups.

A statistically significant difference was found between the two groups in terms of the time taken to reach the maximum sedation and duration of sedation (p < 0.001). The time taken to reach maximum sedation was longer with the MK group (33 ± 9.4 min) than N group (27 ± 3.3min). The duration of sedation as measured from the onset of drug administration till complete recovery and was found to be higher with the MK group (199 ± 24 min) when compared to the N group (87 ± 3 min) (Table 1).

The Houpt’s sedation scale was used for the assessment of sedation in terms of four parameters: sleep, crying, movement, and overall behavior. No statistically significant differences was found between the two groups in terms of any of the parameters assessed (Tables 2 to 5).

The treatment carried out was successful in 80% and 73% of the children in the MK and N groups respectively with no statistically significant differences between the two groups.

No adverse effects were observed in the N group in the present study but in the MK group 6.7% (1 child) reported hallucinations during the sedation procedure, and 20% (3 children) overslept the following night after the procedure as reported by the parent. However, these differences were also not found to be statistically significant.

**Table 1:** **Table 1:** Comparison of the time taken to reach maximum sedation and the anesthesia duration between the groups

		*Group*		*Group*		*N*		*Mean*		*SD*		*Min.*		*Max.*		*t value*		*p value*	
Maximum sedation		MK		15		33.00		9.411		20		50		4.819		0.037			
		N		15		27.33		3.374		21		30							
		Total		30		30.17		7.520		20		50							
Anesthesia duration		MK		15		198.67		23.790		160		245		322.044		< 0.001			
		N		15		87.33		3.374		81		90							
		Total		30		143.00		59.028		81		245							

(Sedation effectiveness as determined by the Houpt’s sedation scale)

**Table 2:** **Table 2:** Comparison of sleep scores between the groups

				*Sleep scale*									
*Group*		*Drowsy*		*Asleep*		*Deep sleep*		*Total*		***χ^2^****value*		*p-value*	
MK		5 33.3%		9 60.0%		1 6.7%		15 100.0%					
N		1 6.7%		12 80.0%		2 13.3%		15 100.0%		3.429		0.180	
Total		6 20.0%		21 70.0%		3 10.0%		30 100.0%					

**Table 3:** **Table 3:** Comparison of movement score between the two groups

		*Movement scale*													
*Group*		*Violent*		*Continuous*		*Controllable*		*No movement*		*Total*		***χ^2^****value*		*p-value*	
MK		3 20.0%		0 0%		7 46.7%		5 33.3%		15 100.0%					
N		3 20.0%		2 13.3%		6 40.0%		4 26.7%		15 100.0%		2.188		0.534	
Total		6 20.0%		2 6.7%		13 43.3%		9 30.0%		30 100.0%					

**Table 4:** **Table 4:** Comparison of crying scores between the two groups

		*Crying scale*													
*Group*		*Hysterical*		*Continuous*		*Intermittent*		*No Crying*		*Total*		***χ^2^****value*		*p-value*	
MK		0 0%		3 20.0%		6 40.0%		6 40.0%		15 100.0%					
N		1 6.7%		4 26.7%		6 40.0%		4 26.7%		15 100.0%		1.543		0.672	
Total		1 3.3%		7 23.3%		12 40.0%		10 33.3%		30 100.0%					

**Table 5:** **Table 5:** Comparison of the overall behavior between the two groups

		*Overall behavior scale*															
*Group*		*Aborted*		*Fair*		*Good*		*Very good*		*Excellent*		*Total*		*χ^2^* *value*		*p-value*	
MK		3 20.0%		1 6.7%		6 40.0%		4 26.7%		1 6.7%		15 100.0%					
N		4 26.7%		3 20.0%		2 13.3%		5 33.3%		1 6.7%		15 100.0%		3.254		0.516	
Total		7 23.3%		4 13.3%		8 26.7%		9 30.0%		2 6.7%		30 100.0%					

## DISCUSSION

As age is one of the important factors to determine child behavior, subjects in the age group of 3 to 10 years were selected in the study as children above 3 years of age are able to communicate with the operator and respond to verbal commands. Since a large percentage of children in this age group are suffering from early childhood caries, the extent of dental emergencies is relatively greater and frequent. Oral sedation is commonly used in younger age groups. Alternatingly effective use of nitrous oxide-oxygen inhalation sedation is used with advantage in the age group of 3 to 10 years.^16,17^ Nitrous oxide is currently the inhalation agent in routine use for conscious sedation in dental practice.^13^ It is easily titrated, generally acceptable to children.

Intravenous midazolam and ketamine rather than the oral form of the drugs were used in the present study because of the ease of the availability of intravenous drugs against medical prescription and the convenience of unit doses of the injection vials.^18^ The oral formulations of drugs are not readily available and are expensive. Due to the bitter taste of both midazolam and ketamine, it is administered with a flavoring agent. Studies have reported the use of fresh honey,^19^ a flavored grape sus-pension,^20^ sugar-free orange or blackcurrant cordial^21^ to mask the bitter taste of the drugs. In the present study, a flavored mango drink (FROOTI^®^) which was easily available in the market and popular among children was used, which showed a good acceptance among them.

A significant difference was found with the sedation duration between the two groups. Due to the oral administration of the drugs a longer duration was observed with the MK group (198 ± 24 min) than with the N group (87 ± 3 min). This could be attributed to the fact that for the onset of action of an orally administered drug a longer time is required because of its high first-pass metabolism. Studies by Shepherd^22^ and Blain^23^ found the mean procedure duration to be 22.6 and 45.2 respectively with the use of nitrous oxide. In other studies, mean procedure periods for conscious sedation range from 22 to 44 minutes.^24^ One study reports that 87.3% of sessions required < 40 minutes and 95.9% of session required < 60 minutes.^3^ Hence, in the present study the procedures which could be completed within an hour’s duration were included.

Variations in the doses of these drugs in combination have been studied in literature; 0.4 mg/kg of midazolam with 5mg/kg of ketamine 12 and 0.75 mg/kg midazolam orally with 5 mg/kg ketamine.^25^ Darlong^26^ and Ozgul^27^ have successfully described the use of low dose 0.25 mg/kg midazolam with 3 mg/kg ketamine in their studies.

When the time between administration of the drug and the initiation of the dental procedure was measured, a statistically significant difference was found between the groups with a longer time taken by the MK group. In the present study, a period of 30 minutes was given prior to an attempt to commence any treatment as AL-Zahrain^28^ recommended a waiting period of at least 25 to 30 minutes. The time taken for the maximum sedation had an average of around 33 minutes which is in accordance with the study by Mohammad^10^ and Darlong^26^ who found an easier parental separation after 19 minutes. In this study, the mean time taken for nitrous oxide administration was dependent on percentage given which was around 27 minutes. This difference was statistically significant when compared to the oral combination showing that with nitrous oxide the time taken to reach maximum sedation was quicker.

Houpt’s sedation scale was used in the present study due to its reliability, simplicity in data interpretation and frequent successful use in previous studies.^29^ In the present study, an average of 73.3% of the children in both the groups showed intermittent sedation scales and no crying which did not interfere with the treatment. This finding was lower when compared to the study by Funks et al.^30^ where 86% of the children did not weep or only mildly wept. Most of the children (60%) were asleep and could be easily aroused in the MK group. This is in accordance with the study by Mohamed^10^ and Roelofse et al.^31^ wherein 53% and 40% of the children respectively sedated by the MK group were asleep but easily aroused.

Bodily movement of various degrees was observed in the present study ranging from mild to violent movements. The violent movement was seen in 20% of the children in both the groups for whom the treatment was aborted. Movements that did not interfere with the dental procedure was seen in 73.3% of the children of both the groups which was similar to the findings in the study by Roelofse et al.^31^ The movement was controlled with the use of a restraint which ranged from just holding the hands to relieve anxiety to holding the body to enable treatment. No other form of physical restraint was used in the present study.

Final outcome of the treatment was considered to define the success rate in our study. Seventy-three percent and 80% of the pulpectomies and 100% and 70% of the extractions were successful in the MK and N groups respectively and thus showing a high success rate with both the regimens. These findings are similar to the study by Funk et al.^30^ who found an overall success rate of 90% for anxiolysis and behavior in the MK group. In the present study hallucinations and oversleep were the only adverse effects encountered in the MK group which is similar to the results of the study by Babita et al.^32^ who reported nausea and vomiting in 2 patients and Roelfose^31^ who reported vomiting in two children when the oral combination was administered. However, in this study, none of the children had any episodes of vomiting.

## CONCLUSION

The study founds both the sedation regimens to be equally effective in obtaining sedation for children aged between 3 to 10 years. However, clinically greater patient compliance is necessary for the administration of the inhalation agent. With this fact in mind, the operator can choose the sedative agent depending on his preference and experience and also patient acceptance.

## CLINICAL SIGNIFICANCE

In our study, we found that the oral low dose KM combination and nitrous oxide-oxygen inhalation was equally effective for anxiolysis in children. As these combinations are well tolerated, safe, they can be used with benefit in the management of children in dental operatory when used wisely.

## LIMITATIONS

- Small sample size and the inability to blind in the study as different routes of drug administration were studied. However utmost care was taken to prevent any bias by employing a single examiner to note the efficacy of the sedative agent in both the groups.
- Further studies with larger sample sizes are required to emphasize the efficacy of these tested drugs in children to bring about anxiolysis during the dental procedure.

## References

[1] Wilson S (2004). Pharmacological management of the pediatric dental patient. Pediatr Dent.

[2] Peretz B (2002). The use of sedation while treating pediatric dental patient in Isreal. Int. J. Pediatr. Dent.

[3] Council O (2013). Guideline on use of nitrous oxide for pediatric dental patients. Pediatr Dent..

[4] Gronbaek AB, Svensson P, Vaeth M, Hansen I, Poulsen S (2014). A placebo-controlled, double-blind, crossover trial on analgesic effect of nitrous oxide-oxygen inhalation. Int J Paediatr Dent..

[5] Mohamed A, Daabiss, Mohamed H (2011). Dexmedetomidine versus ketamine combined with midazolam;a comparison of anxiolytic and sedative premedication in chidlren. BJMP..

[6] Somri M, Parisinos CA, Kharouba J, Cherni N, Smidt A, Abu Ras Z (2012). Optimising the dose of oral midazolam sedation for dental procedures in children: a prospective, randomized and controlled study. Int J Paediatr Dent..

[7] Shapira J, Kupietzky A, Kadari A, Fuks AB, Holan G (2004). Comparison of oral midazolam with and without hydroxyzine in the sedation of pediatric dental patients. Pediatr Dent..

[8] Ozen B, Malamed SF, Cetiner S, Ozalp N, Ozer L, Altun C (2012). Outcomes of moderate sedation in paediatric dental patients. Aust Dent J.

[9] Barkaran S, Breitbart R, Brenner-Zada G, Feldon M, Assa A, Toledano M (2014). A double-blind, randomised, placebo controlled trial of oral midazolam plus oral ketamine for sedation of children during laceration repair. Emerg Med J.

[10] Rabie ME (2005). Combination of oral ketamine and midazolam versus midazolam alone as a premedication in children undergoing tonsillectomy. AJAIC..

[11] Kupietzky A, Houpt MI (1993). Midazolam: a review of its use for conscious sedation of children. Pediatr Dent..

[12] Golpayegani MV, Dehghan F, Ansari G, Shayeghi S (2012). Comparison of oral Midazolam-Ketamine and Midazolam-Pro-methazine as sedative agents in pediatric dentistry. Dent Res J (Isfahan)..

[13] Mahrotra G, Sambhav J (2014). Nitrous oxide inhalation sedation in Pediatric dentistry: a review. Int J Dent Health Sci..

[14] Gross JB, Bailey PL, Caplan RA, Connis RJ, Cote CJ (2005-2006). Practice guidelines for sedation and analgesia by non-anaesthelogists. Anaesthesiology..

[15] Rai K, Hegde A M, Goel K (2007). Sedation in uncooperative children undergoing dental procedures: a comparative evaluation of midazolam, propofol and ketamine. J Clin Pediatr Dent..

[16] Burton JH, Auble TE, Fuchs SM (1998). Effectiveness of 50% nitrous oxide and 50% oxygen during laceration repair in children. Acad. Emerg Med.

[17] Dum-Russell T, Adair SM, Sams DR, Russell CM, Barenie JT (1993). Oxygen saturation and diffusion hypoxia in children following nitrous oxide sedation. Pediatric Dent..

[18] (2001). Curt goho. Oral midazolam-grape fruit juice drug interactin. Ped. Dent..

[19] Darlong V, Shende D, Subramanyam MS, Sunder R, Naik A (2004). Oral ketamine or midazolam or low dose combination for premedication in children. Anaesth Intensive Care..

[20] Silver T, Wilson C, Webb M (1994). Evaluation of two dosages of oral midazolam as a conscious sedation for physically and neurologically compromised pediatric dental patients. Pediatr Dent..

[21] Wilson KE, Girdler NM, Welbury RR (2003). Randomized, controlled, cross-over clinicalt rial comparing intravenous midazolam sedation with nitrous oxide sedation in children undergoing dental extractions. Br J Anaesth..

[22] Shepherd AR, Hill FJ (2000). Orthodontic extractions: a comparative study of inhalation sedation and general anaesthesia. Br Dent J.

[23] Blain KM, Hill FJ (1998). The use of inhalation sedation and local anaesthesia as an alternative to general anaesthesia for dental extractions in children. Br Dent J.

[24] Lyratzopoulos G, Blain KM (2003). Inhalation sedation with nitrous oxide as an alternative to dental general anaesthesia for children. J Public Health Med..

[25] Cagiran E, Eyigor C, Sipahi A, Koca H, Balcioglu T, Uyar M (2010). Comparison of oral Midazolam and Midazolam-Ketamine as sedative agents in paediatric dentistry. Eur J Paediatr Dent..

[26] Darlong V, Shende D, Singh M, Garg R, Pandey R, Punj J (2011). Low- versus high-dose combination of midazolam-ketamine for oral premedication in children for ophthalmologic surgeries. Singapore Med J.

[27] Baygin O, Bodur H (2010). Isik B Effectiveness of premedication agents administered prior to nitrous oxide/oxygen. Eur J Anaesthesiol..

[28] Al-Zahrani AM, Wyne AH, Sheta SA (2009). Comparison of oral midazolam with a combination of oral midazolam and nitrous oxide-oxygen inhalation in the effectiveness of dental sedation for young children. J Indian Soc Pedod Prev Dent..

[29] Sekerci C, Donmez A, Ate§ Y, Okten F (1996). Oral ketamine premedi-cation in children (placebo controlled double-blind study). Eur J Anaesthesiol..

[30] Funk W, Jakob W, Riedl T, Taeger K (2000). oral preanesthetic medication for children: double -blind randomized study of a combination of midazolam and ketamine vs midazolam or ketamine alone. Br J Anaesth..

[31] Roelofse JA, Louw LR, Roelofse PG (1998). A double blind randomized comparison of oral trimeprazine-methadone and ketamine-midazolam for sedation of pediatric dental patients for oral surgical procedures. Anesth Prog..

[32] Babita G, Radhika Prasad G (2005). Comparative evaluation of midazolam and ketamine with midazolam alone as oral premedication. Pediatr Anesth..

